# In Memoriam—Samuel Swan, M. D.

**Published:** 1893-12

**Authors:** Harlyn Hitchcock


### IN MEMORIAM—SAMUEL SWAN, M. D.

Samuel Swan, M. D., was born at Medford, Mass., July 4th, 1815. Died, New York, October 18th, 1893. He was the son of Samuel Swan, a sea captain, who was lost at sea. His grandfather was a major in the Revolutionary war under General Lincoln. Thus we find the Doctor to be of good old American stock, and the old homestead still stands, a relic of colonial days. He attended the Bradford Academy and early developed a talent for music. He entered into the music business and also published a number of his own compositions. He was married in 1843, and as his health was not good went to Montgomery, Ala., and entered mercantile life. During the ten years he was there he became intimate with Dr. Uhlric and Dr. Albright, local physicians, who were practitioners of Homœopathy, and spent much of his spare time in the study of medicine under their direction. He was fortunate in being directed to that line of investigation through the influence of his uncle, Dr. Daniel Swan, of Medford, who as early as 1808 embraced Homœopathy, and was one of the pioneers in the practice in this country.

During the Doctor's residence in Montgomery the yellow fever broke out and a severe epidemic ensued. He did not turn away, but devoted his whole time to caring for the afflicted, acting both as nurse and physician at a time when such assistance was most difficult to obtain. His success was marked, not only as a physician, but in business as well, and he rapidly accumulated a competency. In 1860 he removed to Wilmington, Del., and while there attended the Homœopathic Medical College of Pennsylvania at Philadelphia, from which he was graduated in 1866. In 1863, however, he came to New York, where he made his home till his death.

During his early residence in New York he was associated with Dr. E. Bayard, and their relations were always of the most friendly nature.

Dr. Swan became most widely known through his work in connection with the preparation of high potencies and the use of the morbific products of diseases as remedies. He was interested in the proving of remedies for many years before he began the manufacture of the potencies, which was about the year 1875. With him originated the term "bottle washing," which became the sneer of those who scoffed at the idea of the high potencies, and the excessively high notation which he adopted caused very severe criticism of his method.

In 1881 he became a member of the then newly-organized International Hahnemannian Association, and was one of its most staunch supporters, although in 1887 he severed his connection with the Association because of the most unpleasant complications arising from the use of his membership seal on his catalogue of remedies.

In private life Dr. Swan was one of the most genial and kindly men, beloved and respected by all who knew him. His charities were broad and unostentatious, giving freely of his means and services to those whose need appealed to him, and for five years gave his service free to all who came. Broad and liberal-minded in all things, his religion was as broad as his charities, and held the brightest hopes for all.

By the death of Dr. Swan the profession loses one of its most conscientious students; his widow, son, and daughter a loving husband and father; while his associates and patients mourn a kind and valued friend.

The time has not yet arrived when a just estimate of the work of Dr. Swan can be made. Labors which have extended through years, intermingled with the tumult and turmoil of battle, the sounds of which, together with the heat and passions of conflict are not yet passed away, cannot be considered with the calm and dispassionate judgment that will be accorded them in later days.

Even the bitterest opponent of Dr. Swan cannot but admit his sincerity and earnestness of purpose. If he was in error in his conclusions, the error was of the head and not of the heart. The writer knew him many years and never knew him to utter an unkind word or speak disparagingly of even his most bitter opponent. He simply said in substance, they could not understand his meaning; and that, unfortunately, was one of the difficulties of his methods of expressing himself in writing. He took too much for granted, believing that his readers understood his position and could understand what he intended to convey without further explanation. This the writer has observed a number of times, when a personal interview would give a distinct, and sometimes, almost opposite meaning to something which he had written, and which had been construed in an entirely different manner.

To be sure, it will be necessary to depend upon what he has written, to assign him to his place in homœopathic literature. How much of fact and how much of fancy there is in his investigations and compilations remains for the future to determine. All that he has done must be proved and accepted, or must be disproved and rejected ; it cannot and will not be cast aside because some, however well meaning, have stamped it with their disapproval. Homœopathy will not stand or fall by any one's *dictum*. Its foundation is on facts and on facts depend its existence.

The intense opposition which was developed by his advocacy of the use of the morbific products, reached its culmination when he issued his famous *Catalogue of Morbific Products, Nosodes, and other Remedies, in High Potencies.* There can be no question as to the use of morbific products in potentiated form, any more than there can be a question concerning the use of other products of animal or vegetable origin in like manner and under the Hahnemannian principles. If Psorinum, Syphilinum, Medorrhinum, etc., are legitimate remedies, Morbilin or Pyrogen are equally so. It is to Dr. Swan that we are indebted for many of these agents, and to his indomitable perseverance in the face of the most strenuous opposition that provings of them have been obtained.

With all this, however, the question may be legitimately raised, and without casting any reflections upon his sincerity or honesty of purpose, as to whether he was justified in his methods of using and introducing his "specifics," as he chose to call them. In all probability, it was this, rather than the preparations themselves, which caused his *confréres* to oppose him so strongly. That he believed his oft-repeated generalization, "Morbific matter will cure the disease which produced it, if given in a high potency, even to the person from whom it was obtained," cannot be disputed : and his experience in the use of these products, extending over many years, only served to confirm his belief. The very fact that his observations have been confirmed in hundreds of instances by other physicians—that hundreds of physicians have used his remedies to a greater or less extent, and in many cases with the most surprising and satisfactory results—is sufficient cause for reflection and consideration rather than for hasty condemnation because the generalization was apparently "unorthodox."

The indiscriminate use of the morbific products was never advocated by Dr. Swan to the writer's knowledge. He was a firm believer in the Hahnemannian principles, but like many others, he was satisfied that the homœopathic materia medica was incomplete and would remain incomplete till it embraced everything that could affect the living organism. As Hahnemann found his first provings by the action of the crude drug, so Swan found the first provings of morbific matter by the action of the crude disease-element as shown in the disease condition, and his argument was: If the sum total of all the symptoms arising from any specific disease-producing element can be obtained, we shall have the complete proving of that element. While this proposition is logically correct, the conclusion that the morbific product of the diseased condition contained the disease element, is not strictly true, for while this specific sick-making element does obtain, as may be proved by the process of inoculation, it also contains other elements of varying characteristics according to the source of the morbific matter, hence even the potentiated product is not pure and does not represent the true sick-making element in the original state.

Herein lies the difficulty in the use of the morbific products. That any morbific product will produce the exact totality of symptoms produced by the original disease-producing factor cannot be substantiated, as it is a compound factor in itself: hence it cannot be considered as an *idem* or the same, but being of nosodic origin is still the *homoion* or similar. Therefore, while there cannot be any reasonable objection to the use of remedies of nosodic origin, any more than against those of isotic, organic, or dynamic origin, they must be invariably subjected to the process of proving, according to well-established homœopathic principles.

The small volume, *A Materia Medica*, which Dr. Swan published in 1888, containing the provings of Saccharum-lactis and Lac-caninum, stands in evidence of his industry and labor. This work was never completed because the demand for the work did not warrant the necessary expenditure, and unfortunately, the greater portion of this edition was destroyed by fire and the work is now out of print. It is to be hoped that the large amount of MSS. which the Doctor has left, containing the provings and memoranda of his various remedies may be presented to the profession at no distant day, for they are far too valuable to be lost. HITCHCOCK.

